# High Prevalence of Hepatitis B in People With HIV in the MWCCS, 2018–2024

**DOI:** 10.1093/ofid/ofag346

**Published:** 2026-06-03

**Authors:** Maria F Sanes Guevara, Ken S Ho, Anandi N Sheth, Andrew Edmonds, Audrey L French, David B Hanna, Heather King, Jennifer C Price, Maria L Alcaide, Matthew J Mimiaga, Michael Augenbraun, Michael Plankey, Stephen J Gange, Valentina Stosor, Bernard J C Macatangay, Eric C Seaberg, Phyllis C Tien, Chloe L Thio, Yijia Li, Multicenter AIDS Cohort Study (MACS)/Women's Interagency HIV Study (WIHS) Combined Cohort Study (MWCCS), Cecile Lahiri, Anandi Sheth, Gina Wingood, Todd Brown, Joseph Margolick, Kathryn Anastos, David Hanna, Anjali Sharma, Deborah Gustafson, Tracey Wilson, Gypsyamber D’Souza, Stephen Gange, Elizabeth Topper, Mardge Cohen, Audrey French, Ryan Ross, Steven Wolinsky, Frank Palella, Valentina Stosor, Bradley Aouizerat, Jennifer Price, Phyllis Tien, Roger Detels, Matthew Mimiaga, Seble Kassaye, Daniel Merenstein, Maria Alcaide, Margaret Fischl, Deborah Jones, Jeremy Martinson, Charles Rinaldo, Mirjam-Colette Kempf, James B Brock, Emily Levitan, M Bradley Drummond, Michelle Floris-Moore

**Affiliations:** School of Medicine, University of Pittsburgh, Pittsburgh, Pennsylvania, USA; School of Medicine, University of Pittsburgh, Pittsburgh, Pennsylvania, USA; Division of Infectious Diseases, Department of Medicine, Emory University School of Medicine, Atlanta, Georgia, USA; Department of Epidemiology, University of North Carolina at Chapel Hill, Chapel Hill, North Carolina, USA; Department of Internal Medicine, University of Illinois Chicago, Chicago, Illinois, USA; Department of Epidemiology and Population Health, Albert Einstein College of Medicine, Bronx, NewYork, USA; Division of Infectious Diseases, Department of Medicine, University of Mississippi Medical Center, Jackson, Mississippi, USA; Department of Medicine, University of California SanFrancisco, San Francisco, California, USA; Miller School of Medicine, University of Miami, Miami, Florida, USA; Fielding School of Public Health and David Geffen School of Medicine, University of California Los Angeles, Los Angeles, California, USA; Department of Medicine, SUNY Downstate Health Science University, Brooklyn, NewYork, USA; Department of Medicine, Georgetown University, Washington, DC, USA; Department of Epidemiology, Bloomberg School of Public Health, Johns Hopkins University, Baltimore, Maryland, USA; Feinberg School of Medicine, Northwestern University, Chicago, Illinois, USA; School of Medicine, University of Pittsburgh, Pittsburgh, Pennsylvania, USA; Department of Epidemiology, Bloomberg School of Public Health, Johns Hopkins University, Baltimore, Maryland, USA; Department of Medicine, University of California SanFrancisco, San Francisco, California, USA; Department of Medicine, Johns Hopkins University School of Medicine, Baltimore, Maryland, USA; School of Medicine, University of Pittsburgh, Pittsburgh, Pennsylvania, USA

**Keywords:** antiretroviral therapy, coinfection, HBV, vaccine

## Abstract

**Background:**

This study aimed to examine the prevalence of hepatitis B virus (HBV) in people with HIV (PWH) undergoing contemporary antiretroviral therapy and people without HIV and to understand the risk factors in people with HIV/HBV coinfection. This retrospective cross-sectional study included 5238 participants with HIV or at risk for HIV acquisition across 13 US sites in the MACS/WIHS Combined Cohort Study (Multicenter AIDS Cohort Study / Women Interagency HIV Study) between January 2018 and December 2024.

**Methods:**

The primary outcome was the prevalence of HBV infection and HIV/HBV coinfection. We defined a positive HBsAg (surface antigen) as chronic HBV infection, a negative HBsAg and positive anti-HBV core antibody as recovered infection, and a negative HBsAg and negative anti-HBV core antibody as never infection. The prevalence of chronic and recovered HBV infection was described in different risk groups.

**Results:**

In this cohort, the overall prevalence of chronic HBV was 2.4%, and HIV/HBV coinfection was 3.0% among PWH. Current and prior HBV infection was more common in men, and the prevalence of prior HBV increased with age. As compared with the other 2 groups, PWH with chronic HBV infection had lower CD4+ T-cell counts (567 vs 651 and 689 cells/µL), CD4+ T-cell nadirs (210 vs 245 and 258 cells/µL), and HIV viral suppression rate (71.7% vs 82.3% and 83.7%). Among PWH who never had HBV infection, 40.6% lacked seroprotection against HBV.

**Conclusions:**

HBV is still prevalent in PWH. HBV coinfection was associated with lower CD4+ T-cell counts and viral suppression rate in PWH. The low seroprotection rate in those without HBV underscores the need for improved screening and immunization.

Hepatitis B virus (HBV) infection disproportionately affects people with HIV (PWH) due to shared transmission routes. HIV/HBV coinfection is associated with worse liver-related morbidity and mortality as compared with HBV monoinfection [1, 2]. In the United States, the prevalence of this coinfection was previously estimated to be 5% to 10% based on cohort studies from the 1990s to 2010s [2–4]. However, the landscape of HIV care and prevention has drastically changed in recent years. HIV preexposure prophylaxis, which might include HBV-active agents, is now widely used in populations at risk for HIV. Additionally, the use of dual antiretroviral therapy (ART) regimens with low or no anti-HBV activity—such as dolutegravir/lamivudine, doravirine/islatravir, and injectable cabotegravir/rilpivirine—is gaining popularity, which might increase the risk of HBV reactivation in individuals previously infected and remove the protective effect of tenofovir against new HBV acquisition. Updated HBV vaccination prevents more people from acquiring HBV [5], while rising injection drug use could lead to more HBV acquisition. How these trends may influence HBV prevalence in PWH and people without HIV (PWoH) has not been well studied.

To this end, we leveraged data from the Multicenter AIDS Cohort Study (MACS) / Women Interagency HIV Study (WIHS) Combined Cohort Study (MWCCS) [6], a prospective multicenter cohort study of PWH and at risk for HIV in the United States, to describe the prevalence of HBV infection in PWH and PWoH participating in the cohort between 2018 and 2024. In addition, we aimed to compare different HIV treatment and disease progression markers among HBV serostatus groups and identify risk factors associated with coinfection in this large cohort.

## METHODS

### Study Design, Setting, and Participants

We identified individuals who enrolled and contributed serology testing in the MACS or WIHS or after the merger of the 2 cohorts (MWCCS) between January 2018 and December 2024. If participants had multiple serology tests in this time frame, the first available test since 1 April 2020 (ie, inception of MWCCS) was used; if no MWCCS tests were available, the last test in the historical MACS and WIHS was used. All participants who underwent serologic testing for HBV were included (details in Supplementary Methods) [6]. All participants signed informed consents upon enrollment in the MWCCS. The parent study was approved by local ethical committees from each study site. This study involved only deidentified data from MWCCS data repository and was granted exempt status from the University of Pittsburgh Institutional Review Board.

### Variables

We defined chronic HBV infection as a positive hepatitis B surface antigen, recovered infection as a negative hepatitis B surface antigen with a positive hepatitis B core antibody, and never infection as negative for both markers. HBV immunity was defined as a positive hepatitis B surface antibody. Hepatitis C virus (HCV) exposure was defined as a positive anti-HCV antibody. Detailed demographic and HIV-related parameters are described in the supplementary materials.

### Statistical Methods

We used either χ^2^ tests or Fisher exact tests to compare categorical variables and Kruskal-Wallis tests to compare continuous variables across 3 HBV serogroups. To identify factors associated with HBV infection (chronic and ever), multivariable logistic regression was used. Additional statistical information is included in the supplementary materials. *P* < .05 was deemed statistically significant. R (4.3.1) was used for statistical analysis.

## RESULTS

Among all participants who had follow-up and data collection in this study time frame, we included 5238 of 5263 participants who had complete HBV serology testing. The median age was 54 years (IQR, 44–60); 3560 participants were PWH (68%) and 1678 PWoH (32%). HIV seropositivity was associated with a higher prevalence of chronic HBV infection (PWH 3.0% vs PWoH 1.1%; chronic HBV vs recovered or never, *P* < .001) and recovered HBV infection (PWH 32.7% vs PWoH 27.7%; recovered vs chronic or never, *P* < .001; Table 1). HBV chronic and recovered infection rates were higher in men than women (3.2% vs 1.7% for chronic, 35.0% vs 27.7% for recovered; *P* < .001). In a multivariate analysis adjusting for confounding variables, sex and HIV coinfection remained significantly associated with chronic or recovered HBV infection (Supplementary Table 1). Chronic HBV infection was higher in the 40- to 49-year age group, while the prevalence of recovered HBV infection increased with age. Among participants with chronic HBV infection, 45.2% were men who had sex with men (*P* < .001; Table 1). Recovered HBV infection was associated with substance use, including injection drug use, and HCV exposure as compared with the other subgroups.

**Table 1.** Demographic Information in All Participants Stratified by HBV Serology Status

	Prevalence, No. (%) or Median (IQR)				
Characteristic	Total (n = 5238)	HBV Chronic (n = 126)	HBV Recovered (n = 1628)	HBV Never (n = 3484)	*P* Value ^a^
**Demographics** ^b^					
Age group, y					<.001
20–39	810 (100.0)	15 (1.9)	72 (8.9)	723 (89.3)	
40–49	1118 (100.0)	33 (3.0)	238 (21.3)	847 (75.8)	
50–59	1882 (100.0)	46 (2.4)	634 (33.7)	1202 (63.9)	
≥60	1428 (100.0)	32 (2.2)	684 (47.9)	712 (49.9)	
Age, y	54 (44–60)	53 (44–60)	58 (51–64)	51 (41–58)	<.001
Sex assigned at birth					<.001
Female	2832 (100.0)	48 (1.7)	785 (27.7)	1999 (70.6)	
Male	2406 (100.0)	78 (3.2)	843 (35.0)	1485 (61.7)	
Race and ethnicity					<.001
AAPI	68 (100.0)	2 (2.9)	17 (25.0)	49 (72.1)	
African American	2611 (100.0)	81 (3.1)	806 (30.9)	1724 (66.0)	
Caucasian	1110 (100.0)	19 (1.7)	417 (37.6)	674 (60.7)	
Hispanic or Latino	838 (100.0)	11 (1.3)	211 (25.2)	616 (73.5)	
Other/multiple/unknown	611 (100.0)	13 (2.1)	177 (29.0)	421 (68.9)	
HIV status					<.001
Person without HIV	1678 (100.0)	19 (1.1)	465 (27.7)	1194 (71.2)	
Person with HIV	3560 (100.0)	107 (3.0)	1163 (32.7)	2290 (64.3)	
Region					<.001
Northeast	1287 (100.0)	10 (0.8)	446 (34.7)	831 (64.6)	
South	2514 (100.0)	81 (3.2)	681 (27.1)	1752 (69.7)	
West	780 (100.0)	15 (1.9)	267 (34.2)	498 (63.8)	
Midwest	657 (100.0)	20 (3.0)	234 (35.6)	403 (61.3)	
**Risk factors and characteristics** ^c^					
No. of lifetime sex partners	27 (10–69)	23 (9–68)	36 (11–105)	25 (10–58)	<.001
Unknown	607	14	211	382	
MSM ^d^	1682 (32.1)	57 (45.2)	613 (37.7)	1012 (29.0)	<.001
Housing					<.001
Stable housing	4468 (85.3)	108 (85.7)	1317 (80.9)	3043 (87.3)	
Unstable housing	371 (7.1)	10 (7.9)	142 (8.7)	219 (6.3)	
Other/unknown	399 (7.6)	8 (6.3)	169 (10.4)	222 (6.4)	
Drug use	3321 (63.4)	73 (57.9)	1183 (72.7)	2065 (59.3)	<.001
IDU	313 (6.1)	2 (1.6)	151 (9.5)	160 (4.7)	<.001
IDU status unknown	131	1	38	92	
Income level					.003
Low income	2616 (51.4)	61 (49.6)	867 (54.9)	1688 (49.8)	
Unknown	150	3	50	97	
Insurance status					.004
Insured	4690 (90.4)	114 (90.5)	1489 (92.4)	3087 (89.5)	
Unknown	52	0	17	35	
HBV surface antibody					NA
Negative	2001 (38.3)	126 (100.0)	264 (16.3)	1611 (46.3)	
Positive	3167 (60.6)	0 (0.0)	1340 (82.8)	1827 (52.5)	
Equivocal	57 (1.1)	0 (0.0)	15 (0.9)	42 (1.2)	
Unknown	13	0	9	4	
HCV antibody					<.001
Negative	4504 (87.1)	115 (92.0)	1261 (78.5)	3128 (90.9)	
Positive	665 (12.9)	10 (8.0)	344 (21.4)	311 (9.0)	
Equivocal	4 (0.1)	0 (0.0)	1 (0.1)	3 (0.1)	
Unknown	65	1	22	42	

Abbreviations: AAPI, Asian American and Pacific Islander; HBV, hepatitis B virus; HCV, hepatitis C virus; IDU, intravenous drug use; MSM, men who have sex with men; NA, not applicable.

^a^Categorical variables were compared by either χ^2^ test or Fisher exact test if the prerequisites of the χ^2^ test were not met. For continuous variables, the Kruskal-Wallis test was used.

^b^Row percentage is shown.

^c^Column percentage is shown.

^d^MSM included men who have sex with men only and men who have sex with women and men. Missing data on sex partner number counted as “no MSM.”

When restricting to PWH, chronic HBV infection was similarly more common in men than women (4.2% vs 2.1%), and the prevalence of recovered HBV infection increased with age (Table 2). PWH with chronic HBV infection had a lower median CD4+ T-cell count (567, 653, and 689 cells/µL for chronic, recovered, and never infected, respectively; *P* < .001), CD4 T-cell percentage (31%, 34%, and 36%; *P* < .001), median nadir CD4+ T-cell count (210, 245, and 258 cells/µL; *P* = .002), and HIV viral suppression (71.7%, 83.7%, and 82.3%; *P* = .008; Table 2, Supplementary Figure 1). In the chronic HBV PWH group, only 78.5% of participants received dual HBV-active ART.

**Table 2.** Demographic Information in Participants With HIV Stratified by HBV Serology Status

	Prevalence, No. (%) or Median (IQR)				
Characteristic	Total (n = 3560)	HBV Chronic (n = 107)	HBV Recovered (n = 1163)	HBV Never (n = 2290)	*P* Value ^a^
**Demographics** ^b^					
Age group					<.001
20–39	535 (100.0)	11 (2.1)	58 (10.8)	466 (87.1)	
40–49	806 (100.0)	31 (3.8)	189 (23.4)	586 (72.7)	
50–59	1309 (100.0)	37 (2.8)	469 (35.8)	803 (61.3)	
≥60	910 (100.0)	28 (3.1)	447 (49.1)	435 (47.8)	
Age, y	53 (44–60)	53 (44–61)	57 (51–62)	51 (41–58)	<.001
Sex assigned at birth					<.001
Female	1993 (100.0)	41 (2.1)	575 (28.9)	1377 (69.1)	
Male	1567 (100.0)	66 (4.2)	588 (37.5)	913 (58.3)	
Race and ethnicity					<.001
AAPI	42 (100.0)	1 (2.4)	12 (28.6)	29 (69.0)	
African American	1831 (100.0)	68 (3.7)	602 (32.9)	1161 (63.4)	
Caucasian	664 (100.0)	17 (2.6)	256 (38.6)	391 (58.9)	
Hispanic or Latino	609 (100.0)	8 (1.3)	164 (26.9)	437 (71.8)	
Other/multiple/unknown	414 (100.0)	13 (3.1)	129 (31.2)	272 (65.7)	
Region					<.001
Northeast	787 (100.0)	9 (1.1)	292 (37.1)	486 (61.8)	
South	1758 (100.0)	66 (3.8)	503 (28.6)	1189 (67.6)	
West	570 (100.0)	14 (2.5)	202 (35.4)	354 (62.1)	
Midwest	445 (100.0)	18 (4.0)	166 (37.3)	261 (58.7)	
**Risk factors and characteristics** ^c^					
No. of lifetime sex partners	23 (9–58)	25 (9–71)	31 (10–95)	21 (8–49)	<.001
Unknown	421	13	151	257	
MSM ^d^	1127 (31.7)	47 (43.9)	429 (36.9)	651 (28.4)	<.001
Housing					<.001
Stable housing	3048 (85.6)	92 (86.0)	949 (81.6)	2007 (87.6)	
Unstable housing	238 (6.7)	7 (6.5)	94 (8.1)	137 (6.0)	
Other/unknown	274 (7.7)	8 (7.5)	120 (10.3)	146 (6.4)	
Drug use	2142 (60.2)	64 (59.8)	812 (69.8)	1266 (55.3)	<.001
IDU	206 (5.9)	2 (1.9)	106 (9.3)	98 (4.4)	<.001
IDU status unknown	86	1	24	61	
Income level					.04
Low-income level	1865 (53.7)	56 (53.8)	645 (56.8)	1164 (52.1)	
Unknown	87	3	27	57	
Insurance status					.01
Insured	3212 (90.9)	100 (93.5)	1073 (92.7)	2039 (89.8)	
Unknown	26	0	6	20	
CD4 cell count, cells/µL	673 (463–917)	567 (372–826)	653 (442–882)	689 (475–942)	<.001
Unknown	63	1	29	33	
CD4 cell percentage	35 (27–42)	31 (22–39)	34 (27–41)	36 (28–43)	<.001
Unknown	109	1	40	68	
CD4 count nadir, cells/µL	252 (118–405)	210 (53–334)	245 (117–390)	258 (123–420)	.002
Unknown	6	0	3	3	
HIV viral suppression ^e^					.008
Detectable	615 (17.6)	30 (28.3)	186 (16.3)	399 (17.7)	
Undetectable	2884 (82.4)	76 (71.7)	952 (83.7)	1856 (82.3)	
Unknown	61	1	25	35	
Antiretroviral therapy					.1
TFV + XTC	2495 (70.1)	84 (78.5)	788 (67.8)	1623 (70.9)	
XTC only	604 (17.0)	10 (9.3)	215 (18.5)	379 (16.6)	
TFV only	16 (0.4)	0 (0.0)	8 (0.7)	8 (0.3)	
None or unknown	445 (12.5)	13 (12.1)	152 (13.1)	280 (12.2)	
HBV surface antibody					NA
Negative	1246 (35.1)	107 (100.0)	210 (18.2)	929 (40.6)	
Positive	2261 (63.7)	0 (0.0)	935 (81.0)	1326 (58.0)	
Equivocal	42 (1.2)	0 (0.0)	10 (0.9)	32 (1.4)	
Unknown	11	0	8	3	
HCV antibody					<.001
Negative	3018 (86.0)	98 (92.5)	888 (77.7)	2032 (89.9)	
Positive	489 (13.9)	8 (7.5)	254 (22.2)	227 (10.0)	
Equivocal	2 (0.1)	0 (0.0)	1 (0.1)	1 (0.0)	
Unknown	51	1	20	30	

Abbreviations: AAPI, Asian American and Pacific Islander; HBV, hepatitis B virus; HCV, hepatitis C virus; IDU, intravenous drug use; MSM, men who have sex with men; NA, not applicable; TFV, tenofovir; XTC, emtricitabine or lamivudine.

^a^Categorical variables were compared by either χ^2^ test or Fisher exact test if the prerequisites of the χ^2^ test were not met. For continuous variables, the Kruskal-Wallis test was used.

^b^Row percentage is shown.

^c^Column percentage is shown.

^d^MSM included men who have sex with men only and men who have sex with women and men. Missing data on sex partner number counted as “no MSM.”

^e^Viral load suppression was defined as <50 copies/mL.

Notably, a substantial proportion of participants who never had HBV lacked detectable hepatitis B surface antibody: 46.3% overall and 40.6% among PWH, indicating no evidence of seroprotection. Among those with recovered HBV infection, 16.3% overall and 18.2% of PWH lacked seroprotection (Tables 1 and 2). Among participants who never had HBV infection and who had available self-reported HBV vaccination data (n = 1308), hepatitis B surface antibody seropositivity was associated with self-reported HBV vaccination in the subgroup as a whole and among PWH (positive 57.3%, negative 49.6%, equivocal 55%; *P* = .049) and PWoH (Supplementary Table 2). The vaccination rate was lower as age increased and lower in PWoH than PWH in each age group (Supplementary Figure 2).

In PWoH, we observed similar sex and age distributions for chronic and recovered HBV. In male PWoH, 1.4% had chronic HBV as compared with 0.8% in female. In addition, 57.2% of PWoH who never had HBV infection lacked HBV seroprotection (Supplementary Table 3).

## DISCUSSION

In this large multicenter cohort study of people with or at risk for HIV, we found a chronic HBV infection prevalence of 3.0% in PWH between 2018 and 2024, which is significantly higher than the 1.1% observed in PWoH but lower than historical estimates ranging from 5% to 10% in earlier US-based HIV cohorts [2–4]. This decline may reflect evolving patterns in HIV care and prevention, functional HBV cure in individuals who are coinfected [7], and survival bias from increased mortality in individuals who are coinfected. It also mirrors the broader decline in HBV prevalence observed in the general US population over the past 2 decades [8].

In this cohort, HIV and chronic HBV coinfection was associated with lower current and nadir CD4+ T-cell counts and lower rates of HIV viral suppression as compared with HIV monoinfection. While some studies found no significant differences in virologic suppression by HBV serostatus [9], others reported impaired CD4+ T-cell recovery and suboptimal virologic control among patients who were coinfected [10, 11]. Of note, we observed that almost 20% of PWH with chronic HBV were not receiving HBV-active ART regimens or receiving only partially HBV-active regimens (emtricitabine or lamivudine only) at the time of data collection. This finding is consistent with that from another large HIV/HBV coinfection cohort, where 30% of participants had moderate- to high-risk tenofovir interruptions [12]. Our findings also highlight the need to screen for HBV coinfection in PWH and the necessity of considering HBV status when switching PWH to an HBV-inactive ART regimen [13].

We observed suboptimal rates of HBV immunity among PWH and PWoH who are at risk for HIV and, similarly, HBV acquisition. These data underscore the importance of routine screening for HBV immunity and timely immunization, particularly among individuals prescribed HBV-inactive ART regimens who remain vulnerable to infection [5, 14].

There are several limitations to this study. Given the cross-sectional nature of the study, chronic HBV was defined as only 1 positive hepatitis B surface antigen, which may also represent self-limited acute infection or transient positivity after vaccination. Future studies are warranted to determine incident HBV infection, seroclearance/seroconversion, and immune responses to HBV vaccinations in the modern ART era. Of note, PWoH in this cohort are typically at higher risk for HIV acquisition than the general population; thus, the HBV prevalence in the PWoH group was likely to be higher than in the general population given shared transmission routes between HIV and HBV. Finally, HBV vaccination status was based on self-report, which is subject to recall bias.

## CONCLUSION

In the MWCCS cohort between 2018 and 2024, the prevalence of chronic HBV infection was 3.0% in PWH, a higher proportion than that observed in PWoH. Chronic HBV infection was also higher in males than females. In PWH who never had HBV infection, 40.6% lacked seroprotection against HBV, underscoring the need for improved screening and effective vaccinations.

## Supplementary Material

ofag346_Supplementary_Data

## Supplementary Data

Supplementary materials are available at *Open Forum Infectious Diseases* online. Consisting of data provided by the authors to benefit the reader, the posted materials are not copyedited and are the sole responsibility of the authors, so questions or comments should be addressed to the corresponding author.
