# Supplementary material for: High Prevalence of Hepatitis B in People With HIV in the MWCCS, 2018–2024
Source: Open Forum Infect Dis. 2026 Jun 3;13(6):ofag346. doi: 10.1093/ofid/ofag346 (PMC13280642; doi:10.1093/ofid/ofag346)
Supplement: ofag346_Supplementary_Data [file ofag346_supplementary_data.docx]

**Supplementary materials**

**Title**: High Prevalence of Hepatitis B in People with HIV in the MWCCS, 2018-2024

**Authors:** Maria Sanes Guevara, Ken S. Ho, Anandi N. Sheth, Andrew Edmonds, Audrey L. French, David B. Hanna, Heather King, Jennifer C. Price, Maria L. Alcaide, Matthew J. Mimiaga, Michael Augenbraun, Michael Plankey, Stephen Gange, Valentina Stosor, Bernard JC. Macatangay, Eric C. Seaberg, Phyllis C. Tien, Chloe L. Thio, Yijia Li

Table of Contents

[SUPPLEMENTARY METHODS 2](#_Toc220420397)

[Participants 2](#_Toc220420398)

[Variables 2](#_Toc220420399)

[HBV serology 2](#_Toc220420400)

[HBV vaccination information 2](#_Toc220420401)

[Pairwise multiple comparisons 3](#_Toc220420402)

[Univariate and multivariate logistic regression 3](#_Toc220420403)

[SUPPLEMENTARY TABLES 4](#_Toc220420404)

[Supplementary Table S1. Characteristics associated with chronic HBV and recovered HBV infection in all participants. 4](#_Toc220420405)

[Supplementary Table S2. HBV vaccination status in participants who never had HBV. 6](#_Toc220420406)

[Supplementary Table S3. Demographic information in participants without HIV stratified by Hepatitis B serology status. 7](#_Toc220420407)

[SUPPLEMENTARY FIGURES 9](#_Toc220420408)

[Supplementary Figure S1. CD4 T-cell percent (A), cell count (B), and nadir cell count (C) stratified by hepatitis B serostatus. 9](#_Toc220420409)

[Supplementary Figure S2. Self-reported HBV vaccination status stratified by age group, regardless HBV serostatus. 10](#_Toc220420410)

## **SUPPLEMENTARY METHODS**

### ***Participants***

The Multicenter AIDS Cohort Study (MACS) was a multicenter cohort study enrolling male participants with HIV or at risk for HIV acquisition since 1983. The Women’s Interagency HIV Study (WIHS) was a multicenter cohort study enrolling female participants with HIV or at risk for HIV acquisition since 1993. People with HIV (PWH) and people without HIV (PWoH) in both cohorts shared similar demographics and risk factors. In 2019, MACS and WIHS started to merge into a combined cohort, the MACS/WIHS Combined Cohort Study (MWCCS), and the first MWCCS visit started in late 2020. Approximately 25% of the historic MACS and WIHS participants (n=562) who were rolled over to the MWCCS had hepatitis B (HBV) serology testing in 2020 or later and the rest had the testing done between 2018-2019 (n=2256). In addition, new enrollees (n=1912 in final analysis) in the MWCCS had HBV serology testing. For participants who did not roll over to MWCCS after 2020, 508 had HBV serology testing done between 2018-2019. Informed consent was signed before enrollment and carryover, and the Institutional Review Board from each MWCCS site approved this study.

### ***Variables***

Basic demographic information (e.g., sex, region, any type of drug use except marijuana use, intravenous drug use) was obtained from the contemporaneous visit. For PWH, current and nadir CD4 T-cell count, current CD4 T-cell percentage, HIV viral load, HIV viral load suppression (defined as <50 copies/ml) and ART information were extracted from the visit contemporaneous to the serology test. Additional variables associated with risk for HIV and HBV acquisition, including sexual practice and number of sexual partners, substance use, insurance status, and housing were also extracted.

For participants with multiple HBV testing during the 01/2018-09/2024 timeframe, we prioritized the first available data point since the inception of MWCCS combined cohort. For those who only had data before MACS and WIHS mergers (between 01/2018-09/2024), the latest data point was used.

For drug use information, we extracted available variables on cocaine use (variable names BSCOCUSE, MWBSCOC, MWBSCCH, BSCCHUSE) , heroin and other opiate use (BSHEROPIATEUSE and MWBSHEROPIATE), other illicit drug use including uppers/downers/poppers/methamphetamines/ prescription drug misuse/ other illicit drugs (BSODRGUSE and MWBSODRG). We also extracted a separate variable that reflected injection drug use (BSIDU and MWBSIDU). Marijuana use was excluded. If any of these categories was answered “Yes”, this participant would be categorized as individual with drug use.

### ***HBV serology***

HBV serology was tested using the Enzyme-Linked Immunosorbent Assay according to manufacturer’s instructions.

### ***HBV vaccination information***

Self-reported HBV vaccination information was collected during the MWCCS enrollment.

### ***Pairwise multiple comparisons***

To compare CD4 T-cell percent, cell count, and nadir cell count (Supplementary eFigure 2) in a pairwise fashion after Kruskal-Wallis test, we used Dunn's test with Benjamini-Hochberg adjustment. “rstatix” package was used.

***Missing information***

For HBV status, we included only participants with available hepatitis B surface antigen (HBsAg) results. Among those with negative HBsAg, anti-HBV core antibody (anti-HBc) data was required for classification. For other clinical variables such as CD4 count and HIV viral load, all available values were incorporated, and missing data were explicitly recorded as such. When contemporaneous HIV viral load or CD4 data were not available at the time of HBV serology, we used the measurement closest to that visit within the study period (2018–2024). For HCV status, we used any available result in the database, as a positive HCV antibody remains positive once acquired, and missing data was recorded as such. Immunization status was assessed through a one-time self-reported questionnaire; documented vaccination was recorded when available, and missing data were designated as unknown.

### ***Univariate and multivariate logistic regression***

We performed logistic regression to evaluate association between age, sex, HIV status and HBV status. In univariate analysis, each demographic variable was entered into the Logistic regression formula as an independent variable. HBV chronic infection or ever having HBV infection (both chronic and recovered) was set as dependent variables. We used the ‘glm’ function based on R platform and binomial with logit link. In multivariate analysis, we included HIV status, age group, sex, race/ethnicity, and geographic location to the logistic models. In PWH subgroup, additional covariates including CD4 cell count, CD4 nadir count, and viral suppression were added in the multivariate analysis. To examine the interaction between HIV status, age and sex, additional multivariate models that include age group-HIV status and sex- HIV status interaction terms were examined using the analysis of variance (ANOVA) Wald type 3 test. If P for interaction <0.1, the interaction term would be included in the final multivariate model. R platform was used (Version 4.3.1).

## **SUPPLEMENTARY TABLES**

### **Supplementary Table S1. Characteristics associated with chronic HBV and recovered HBV infection in all participants.**

| **Table S1A. Characteristics associated with chronic HBV infection** | | | | |
| --- | --- | --- | --- | --- |
| **Characteristics** | **Crude OR** **(95% CI)** | **P** | **aOR** **(95% CI)** | **P** |
| Age group |  |  |  |  |
| 20-39 | Ref | NA | Ref | NA |
| 40-49 | 1.61 (0.89 to 3.07) | 0.13 | 2.10 (1.14 to 4.04) | **0.021** |
| 50-59 | 1.33 (0.75 to 2.47) | 0.35 | 1.86 (1.04 to 3.51) | **0.044** |
| Over 60 | 1.21 (0.67 to 2.32) | 0.54 | 1.79 (0.96 to 3.50) | 0.076 |
| Sex assigned at birth |  |  |  |  |
| Female | Ref | NA | Ref | NA |
| Male | 1.94 (1.36 to 2.81) | **<0.001** | 2.55 (1.74 to 3.77) | **<0.001** |
| Race |  |  |  |  |
| Non-Hispanic White/Caucasian | Ref | NA | Ref | NA |
| Non-Hispanic AAPI | 1.74 (0.27 to 6.17) | 0.46 | 2.33 (0.36 to 8.52) | 0.27 |
| Non-Hispanic Black/African American | 1.84 (1.14 to 3.13) | **0.018** | 2.23 (1.31 to 3.95) | **0.004** |
| Hispanic or Latino | 0.76 (0.35 to 1.59) | 0.48 | 0.94 (0.42 to 2.00) | 0.87 |
| Other/Multiple/Unknown | 1.25 (0.60 to 2.52) | 0.54 | 1.46 (0.68 to 3.05) | 0.32 |
| Region |  |  |  |  |
| Northeast | Ref | NA | Ref | NA |
| South | 4.25 (2.31 to 8.77) | **<0.001** | 3.35 (1.79 to 7.00) | **<0.001** |
| West | 2.50 (1.13 to 5.78) | **0.025** | 2.34 (1.05 to 5.46) | **0.041** |
| Midwest | 4.01 (1.91 to 8.98) | **<0.001** | 3.49 (1.65 to 7.84) | **0.001** |
| HIV status |  |  |  |  |
| PWoH | Ref | NA | Ref | NA |
| PWH | 2.71 (1.70 to 4.56) | **<0.001** | 2.55 (1.59 to 4.32) | **<0.001** |
| **Table S1B. Characteristics associated with chronic and recovered HBV infection** | | | | |
| **Characteristics** | **Crude OR** **(95% CI)** | **P** | **aOR** **(95% CI)** | **P** |
| Age group |  |  |  |  |
| 20-39 | Ref | NA | Ref | NA |
| 40-49 | 2.66 (2.06 to 3.47) | **<0.001** | 3.00 (2.31 to 3.94) | **<0.001** |
| 50-59 | 4.70 (3.71 to 6.02) | **<0.001** | 5.34 (4.18 to 6.88) | **<0.001** |
| Over 60 | 8.36 (6.57 to 10.7) | **<0.001** | 9.55 (7.43 to 12.4) | **<0.001** |
| Sex assigned at birth |  |  |  |  |
| Female | Ref | NA | Ref | NA |
| Male | 1.49 (1.33 to 1.67) | **<0.001** | 1.23 (0.98 to 1.56) | 0.077 |
| Race |  |  |  |  |
| Non-Hispanic White/Caucasian | Ref | NA | Ref | NA |
| Non-Hispanic AAPI | 0.60 (0.34 to 1.01) | 0.065 | 1.00 (0.55 to 1.75) | >0.99 |
| Non-Hispanic Black/African American | 0.80 (0.69 to 0.92) | **0.002** | 1.47 (1.23 to 1.76) | **<0.001** |
| Hispanic or Latino | 0.56 (0.46 to 0.68) | **<0.001** | 0.88 (0.71 to 1.09) | 0.25 |
| Other/Multiple/Unknown | 0.70 (0.57 to 0.86) | **<0.001** | 1.21 (0.96 to 1.53) | 0.11 |
| Region |  |  |  |  |
| Northeast | Ref | NA | Ref | NA |
| South | 0.79 (0.69 to 0.91) | **0.001** | 0.85 (0.73 to 0.99) | **0.038** |
| West | 1.03 (0.86 to 1.24) | 0.74 | 1.01 (0.83 to 1.23) | 0.92 |
| Midwest | 1.15 (0.95 to 1.39) | 0.16 | 1.09 (0.88 to 1.34) | 0.42 |
| HIV status |  |  |  |  |
| PWoH | Ref | NA | Ref | NA |
| PWH | 1.37 (1.21 to 1.55) | **<0.001** | 1.20 (1.00 to 1.45) | 0.056 |
| Sex-HIV interaction term |  |  |  |  |
| Male - PWH |  |  | 1.62 (1.24 to 2.12) | **<0.001** |

Abbreviations: AAPI, Asian American and Pacific Islander; CI, confidence interval; HBV, hepatitis B virus; OR, odds ratio; aOR, adjusted odds ratio; PWoH, person without HIV; PWH, person with HIV.

### **Supplementary Table S2. HBV vaccination status in participants who never had HBV.**

| **Whole cohort** | | | | | |
| --- | --- | --- | --- | --- | --- |
| **HBV surface antibody** | **Overall** N = 1,308 | **Negative N = 554** | **Positive** N = 730 | **Equivocal** N = 24 | **P value^a^** |
| Reported ever receiving HBV vaccination, No. (%) | 606 (46.3) | 202 (36.5) | 392 (53.7) | 12 (50.0) | <0.001 |
| **PWH only** | | | | | |
| **HBV surface antibody** | **Overall** N = 870 | **Negative** N = 319 | **Positive** N = 531 | **Equivocal** N = 20 | **P value^a^** |
| Reported ever receiving HBV vaccination, No. (%) | 470 (54.0) | 155 (48.6) | 304 (57.3) | 11 (55.0) | 0.049 |
| **PWoH only** | | | | | |
| **HBV surface antibody** | **Overall** N = 438 | **Negative** N = 235 | **Positive** N = 199 | **Equivocal** N = 4 | **P value^a^** |
| Reported ever receiving HBV vaccination, No. (%) | 136 (31.1) | 47 (20.0) | 88 (44.2) | 1 (25.0) | <0.001 |

Abbreviations: HBV, hepatitis B virus; PWoH, person without HIV; PWH, person with HIV.

^a^ P value was calculated using the Chi-squared test or Fisher’s exact test if prerequisite of Chi-squared test is not met.

### **Supplementary Table S3. Demographic information in participants without HIV stratified by Hepatitis B serology status.**

| **Table S3A. Prevalence of HBV chronic and recovered infection in different demographic groups. Row percentage is shown.** | | | | | |
| --- | --- | --- | --- | --- | --- |
| **Characteristics** | **Overall** N = 1,678 | **HBV Chronic** N = 19 | **HBV Recovered** N = 465 | **HBV Never** N = 1,194 | **P values^a^** |
| Age group, No. (%) |  |  |  |  | <0.001 |
| 20-39 | 275 (100.0) | 4 (1.5) | 14 (5.1) | 257 (93.5) |  |
| 40-49 | 312 (100.0) | 2 (0.6) | 49 (15.7) | 261 (83.7) |  |
| 50-59 | 573 (100.0) | 9 (1.6) | 165 (28.8) | 399 (69.6) |  |
| Over 60 | 518 (100.0) | 4 (0.8) | 237 (45.8) | 277 (53.5) |  |
| Median age, years (IQR) | 54 (44, 62) | 52 (41, 59) | 60 (54, 66) | 51 (42, 59) | <0.001 |
| Sex assigned at birth, No. (%) |  |  |  |  | 0.021 |
| Female | 839 (100.0) | 7 (0.8) | 210 (25.0) | 622 (74.1) |  |
| Male | 839 (100.0) | 12 (1.4) | 255 (30.4) | 572 (68.2) |  |
| Race/Ethnicity, No. (%) |  |  |  |  | <0.001 |
| AAPI | 26 (100.0) | 1 (3.8) | 5 (19.2) | 20 (76.9) |  |
| African American | 780 (100.0) | 13 (1.7) | 204 (26.2) | 563 (72.2) |  |
| Caucasian | 446 (100.0) | 2 (0.4) | 161 (36.1) | 283 (63.5) |  |
| Hispanic or Latino | 229 (100.0) | 3 (1.3) | 47 (20.5) | 179 (78.2) |  |
| Other/Multiple/Unknown | 197 (100.0) | 0 (0.0) | 48 (24.4) | 149 (75.6) |  |
| Region, No. (%) |  |  |  |  | 0.002 |
| Northeast | 500 (100.0) | 1 (0.2) | 154 (30.8) | 345 (69.0) |  |
| South | 756 (100.0) | 15 (2.0) | 178 (23.5) | 563 (74.5) |  |
| West | 210 (100.0) | 1 (0.5) | 65 (31.0) | 144 (68.6) |  |
| Midwest | 212 (100.0) | 2 (0.9) | 68 (32.1) | 142 (67.0) |  |
| **Table S3B. Prevalence of certain risk factors and characteristics in different HBV serostatus groups. Column percentage is shown.** | | | | | |
| **Characteristics** | **Overall** N = 1,678 | **HBV Chronic** N = 19 | **HBV Recovered** N = 465 | **HBV Never** N = 1,194 | **P values^a^** |
| Number of lifetime sex partners, median (IQR) | 36 (15, 95) | 21 (8, 60) | 49 (17, 146) | 34 (14, 79) | <0.001 |
| Unknown | 186 | 1 | 60 | 125 |  |
| MSM^b^, No. (%) | 555 (33.1) | 10 (52.6) | 184 (39.6) | 361 (30.2) | <0.001 |
| Housing, No. (%) |  |  |  |  | 0.001 |
| Stable housing | 1,420 (84.6) | 16 (84.2) | 368 (79.1) | 1,036 (86.8) |  |
| Unstable housing | 133 (7.9) | 3 (15.8) | 48 (10.3) | 82 (6.9) |  |
| Unknown housing | 125 (7.4) | 0 (0.0) | 49 (10.5) | 76 (6.4) |  |
| Drug use, No. (%) | 1,179 (70.3) | 9 (47.4) | 371 (79.8) | 799 (66.9) | <0.001 |
| IDU, No. (%) | 107 (6.6) | 0 (0.0) | 45 (10.0) | 62 (5.3) | 0.003 |
| IDU status Unknown | 45 | 0 | 14 | 31 |  |
| Income level, No. (%) |  |  |  |  |  |
| Low income | 751 (46.5) | 5 (26.3) | 222 (50.2) | 524 (45.4) | 0.047 |
| Unknown | 63 | 0 | 23 | 40 |  |
| Insurance status, No. (%) |  |  |  |  |  |
| Insured | 1,478 (89.5) | 14 (73.7) | 416 (91.6) | 1,048 (88.9) | 0.025 |
| Unknown | 26 | 0 | 11 | 15 |  |
| HBV surface antibody, No. (%) | |  |  |  | NA |
| Negative | 755 (45.0) | 19 (100.0) | 54 (11.6) | 682 (57.2) |  |
| Positive | 906 (54.1) | 0 (0.0) | 405 (87.3) | 501 (42.0) |  |
| Equivocal | 15 (0.9) | 0 (0.0) | 5 (1.1) | 10 (0.8) |  |
| Unknown | 2 | 0 | 1 | 1 |  |
| HCV antibody, No. (%) |  |  |  |  | <0.001 |
| Negative | 1,486 (89.3) | 17 (89.5) | 373 (80.6) | 1,096 (92.7) |  |
| Positive | 176 (10.6) | 2 (10.5) | 90 (19.4) | 84 (7.1) |  |
| Equivocal | 2 (0.1) | 0 (0.0) | 0 (0.0) | 2 (0.2) |  |
| Unknown | 14 | 0 | 2 | 12 |  |
| Abbreviations: AAPI, Asian American and Pacific Islander; HBV, hepatitis B virus; HCV, hepatitis C virus; IDU, intravenous drug use; IQR, interquartile range; MSM, men who have sex with men.  ^a^ P value was calculated using the Chi-squared test or Fisher’s exact test if prerequisite of Chi-squared test is not met.  ^b^ MSM counted both men who have sex with men only and men who have sex with women and men. Missing data on sex partner number since Visit 60 counted as No MSM. | | | | | |

## **SUPPLEMENTARY FIGURES**


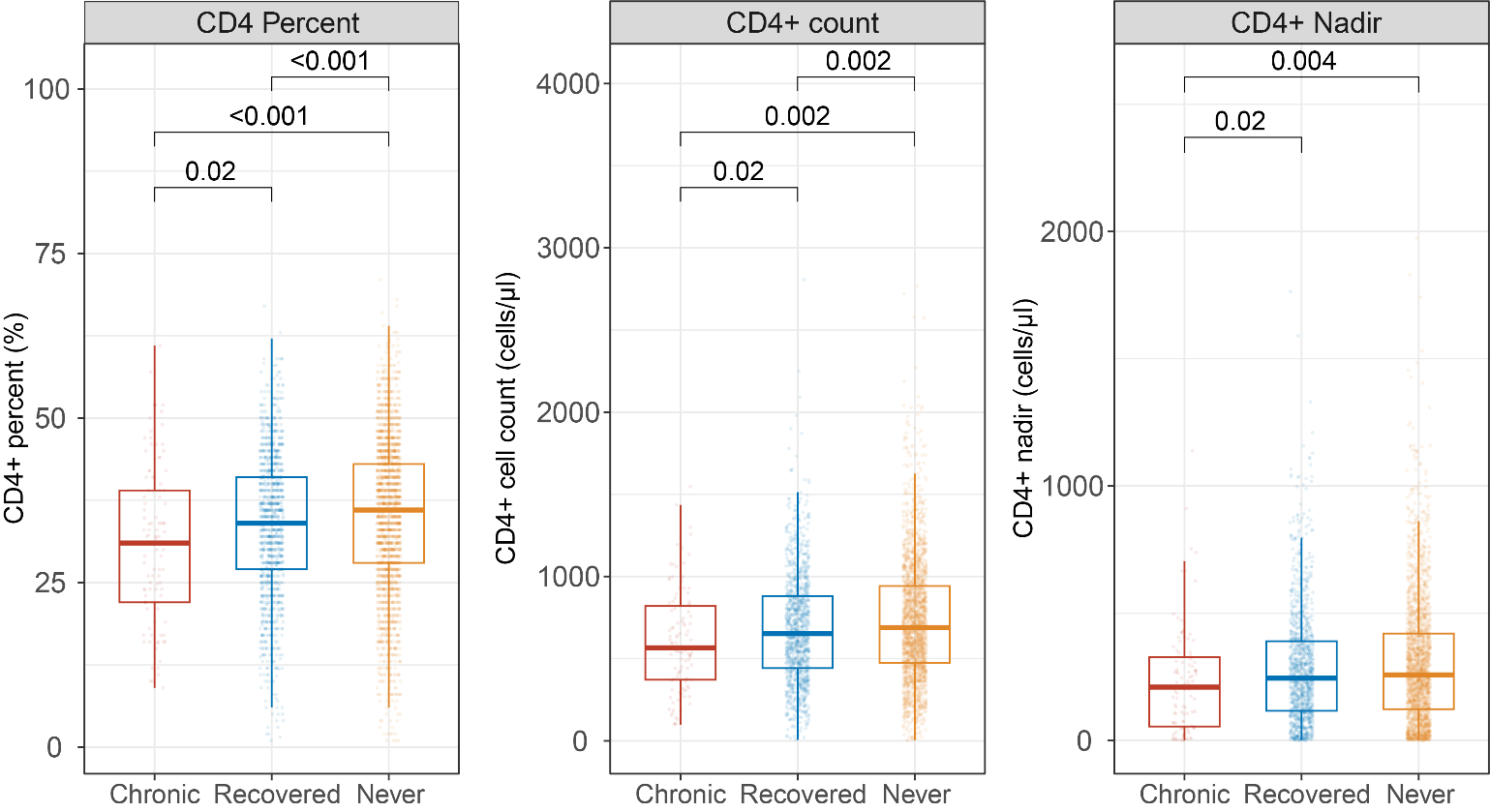


### **Supplementary Figure S1. CD4 T-cell percent (A), cell count (B), and nadir cell count (C) stratified by hepatitis B serostatus.**

P values were calculated using Dunn’s test and Benjamini-Hochberg adjustment. Tukey boxplot with individual values (dots) are shown. The middle horizontal line in the boxplot represents the median and the box limits represent the first and third quartiles (Q1 and Q3). The lower whisker is the minimum value within 1.5 times the interquartile range (IQR) under Q1, while the upper whisker is the maximum value within 1.5 times the interquartile range (IQR) over Q3.


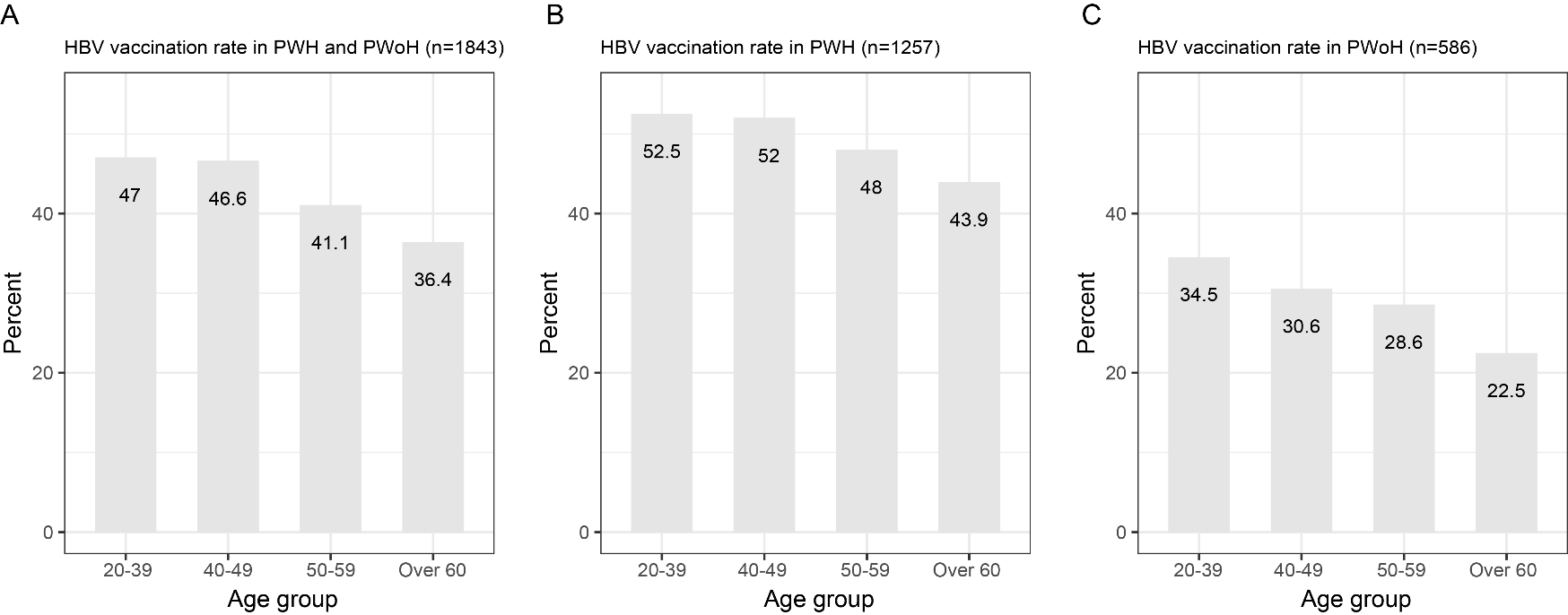


### **Supplementary Figure S2. Self-reported HBV vaccination status stratified by age group, regardless HBV serostatus.**

HBV vaccination rate in (A) PWH and PWoH; (B) PWH; (C) PWoH. Of note only 1,843 participants had available HBV vaccination information based on self-report. HBV, hepatitis B virus; PWH, person with HIV; PWoH, person without HIV.
